# An Unusual Case of Mucinous Adenocarcinoma of the Lung Presenting as Septic Emboli

**DOI:** 10.7759/cureus.40326

**Published:** 2023-06-12

**Authors:** Brinn DeMars, Mohammad Khan, David P Lebel, Badri Giri

**Affiliations:** 1 Internal Medicine, Virginia Tech Carilion School of Medicine, Roanoke, USA; 2 Pulmonary and Critical Care, Virginia Tech Carilion School of Medicine, Roanoke, USA; 3 Basic Science Education, Virginia Tech Carilion School of Medicine, Roanoke, USA

**Keywords:** mucinous adenocarcinoma, cavitary lung lesion, nonbacterial thrombotic endocarditis, septic pulmonary emboli, adenocarcinoma lung

## Abstract

This is a case of a 92-year-old female with multiple hospitalizations for dyspnea on exertion and hypoxemia. Her symptoms were initially thought to be secondary to pneumonia, and on subsequent admission, culture-negative endocarditis. A computed tomography (CT) of the chest was remarkable for numerous bilateral lung nodules of varying size, some of which had a cavitary appearance raising concern for septic emboli. While a transthoracic echo was unremarkable, a transesophageal echo found a small 3 mm echodensity at the tip of the right coronary leaflet of the aortic valve and a possible mobile echodensity on the tricuspid valve leaflet. These findings further supported a clinical diagnosis of endocarditis with septic emboli in the lungs. Initial bronchoscopy yielded an unremarkable biopsy and a bronchial alveolar lavage with the growth of *Actinomyces odontolyticus*. During a subsequent hospitalization, a repeat bronchoscopy with transbronchial biopsy revealed a final diagnosis of invasive pulmonary mucinous adenocarcinoma. This case highlights a unique presentation of mucinous adenocarcinoma of the lung initially masquerading as septic emboli, resulting in a delay in the final diagnosis.

## Introduction

This case was previously presented at the Virginia ACP Residents Meeting and Poster Competition on 10/22/2022. Lung cancer is one of the most frequently diagnosed malignancies and is the second leading cause of death in both men and women [1]. It is responsible for nearly a quarter of cancer deaths. In fact, lung cancer is attributable to more deaths than breast, prostate, and colorectal cancer combined [1]. Most diagnoses are related to tobacco use, yet there is still a substantial number of lung cancer diagnoses in non-smokers [1]. The incidence in men has been declining over several years compared to women, which is thought to be secondary to decreased smoking in men and possibly increased smoking in women [1]. Five-year survival remains quite poor which is largely attributed to the presence of advanced clinical stage at the time of diagnosis owing to the relatively asymptomatic initial course of the disease [1]. Screening initiatives such as the 2013 implementation of low-dose computed tomography (CT) screening are likely to result in earlier detection and treatment of high-risk patients [1]; however, such modalities do not capture low-risk populations and cannot assess asymptomatic patients. Treatment has also improved resulting in a decrease in mortality as lung cancer mortality was responsible for a reduction in nearly 50% of the overall cancer mortality between 2014 and 2018 [1].

Non-bacterial thrombotic endocarditis (NBTE) occurs when thrombi develop on heart valves without the presence of bacteremia. The incidence of NBTE is uncertain as it is a rare condition, and the diagnosis is often found post-mortem. In a large autopsy case series, 65 cases of NBTE were discovered; of these, 51 such cases were associated with malignancy [2]. The hypercoagulable state of malignancy is a known risk factor for thromboembolic events. Specifically, pancreatic, brain, renal, lung, and ovarian cancer have all been associated with a high risk for venothromboembolic events [3]. The aortic and mitral valves are most commonly affected in NBTE, while the involvement of right-sided heart valves is less common [2].

Herein, we present a case of a primary pulmonary mucinous adenocarcinoma clinically masquerading as endocarditis with septic emboli to highlight an unusual clinical manifestation in hopes of impacting earlier detection and diagnostic intervention.

## Case presentation

The present case took place at a tertiary care medical center in Southwest Virginia. The patient is a 92-year-old female with a past medical history of lupus, prior tobacco use (30-pack-year history), gastroesophageal reflux disease (GERD), obstructive sleep apnea (OSA), and scoliosis who presented with chief complaint of dyspnea on exertion. Her social history was remarkable for living on a farm, but without noted livestock exposures. The patient’s family suspected possible asbestos in the home, but this was not confirmed. No pertinent family history was obtained. A review of systems was remarkable only for an unspecified amount of weight loss over the prior few months, a dry cough, fatigue, back pain, and a poor appetite.

She was initially hospitalized in October of 2021 at an outside facility for presumed pneumonia and treated with levofloxacin. The radiology report of a CT scan performed at the same outside facility was remarkable for pneumonia and bilateral lung nodules suspicious for septic emboli. Due to persistent symptoms, she was prescribed doxycycline and itraconazole by her primary care physician.

She was again hospitalized in December 2021 for two months of nonproductive cough and hypoxia on exertion. During this hospitalization, this patient remained afebrile and hemodynamically stable without leukocytosis. Oxygen saturations were maintained on room air. Initial arterial blood gas obtained on presentation was notable for a pO2 of 69, pCO2 of 31, and bicarbonate of 20.5 but otherwise was within normal limits.

**Figure 1:**
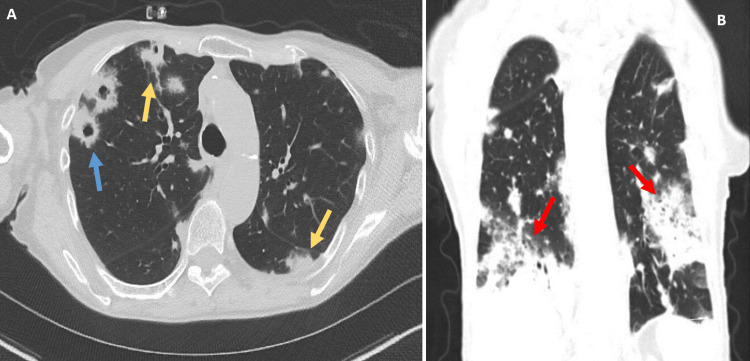
CT of the Chest Findings A: varying-sized nodules, some of which are cavitary (blue arrow) with ground glass opacities (yellow arrows). B: coronal view of more consolidated lesions and nodules (red arrows) with bilateral distributions. CT: computed tomography

A CT scan was remarkable for bilateral lung nodules of various sizes, some of which appeared cavitary and were again thought to be consistent with septic emboli (Figure 1). Consolidations were also present in the bilateral lower lobes raising suspicion of superimposed pneumonia. The differential diagnosis at that time included septic emboli versus hematogenous spread of metastatic malignancy. A transthoracic echocardiogram showed preserved ejection fraction without any vegetation. A transesophageal echo demonstrated a small 3 mm echodensity at the tip of the right coronary leaflet of the aortic valve and a possible mobile echodensity on the tricuspid valve leaflet. These findings were suggestive of vegetation and a potential source for the suspected septic emboli. Bronchoscopy was performed and cultures from a bronchoalveolar lavage grew *Actinomyces odontolyticus*. Transbronchial biopsies from the same bronchoscopy were unremarkable.

*Actinomyces *spp. is typically found within the oropharynx, and lung infections are often the result of pulmonary aspiration. The patient was seen with an infectious disease during this hospitalization. Antibiotics were not initially prescribed on discharge and a workup for culture-negative endocarditis was initiated. However, the patient followed up with infectious disease as an outpatient and a three-month course of amoxicillin were started to address the *Actinomyces *infection despite low clinical suspicion for infection.

**Figure 2:**
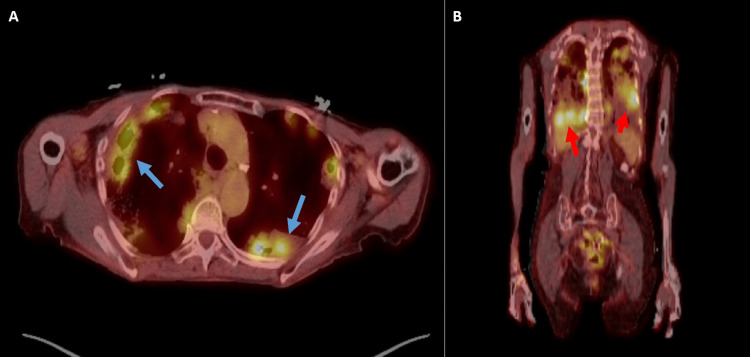
PET-CT Findings A: multiple pulmonary nodules demonstrating increased radiotracer uptake (blue arrows). B: pulmonary nodules and areas of consolidation with increased avidity (red arrows). PET-CT: positron emission tomography-computed tomography

**Figure 3:**
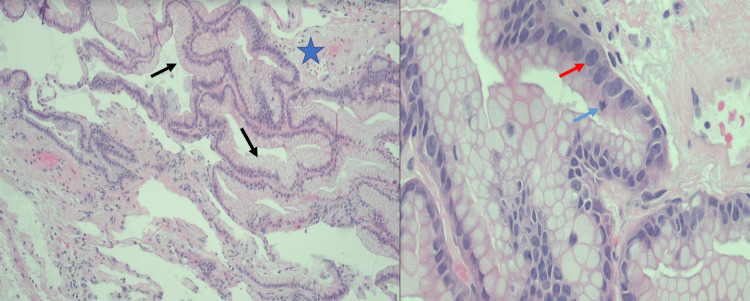
Transbronchial Biopsies A: Hematoxylin and eosin-stained slide from the transbronchial biopsies showing the native alveolar spaces (star) lined by an atypical mucinous epithelium (arrows) showing apparent abrupt onset from the normal alveolar spaces in the bottom left. B: A higher power photomicrograph demonstrating the mucinous epithelium with mild to moderate atypia including nuclear enlargement, hyperchromasia (red arrow), and few identifiable mitoses (blue arrow).

The patient was again hospitalized for a third time in March of 2022 with worsening dyspnea and hypoxia. CT scan at that time again revealed the persistence of the bilateral pulmonary nodules and consolidations. Positron emission tomography (PET) CT was also obtained and significant for multiple bilateral avid pulmonary abnormalities suggestive of malignancy, infection, or inflammation (Figure 2). She was again started on antibiotics and a clinical workup for rheumatologic or autoimmune processes ensued. The differential diagnosis at this time included septic emboli, Libman-Sacks endocarditis, and malignancy. She again underwent bronchoscopy with bronchoalveolar lavage and transbronchial biopsies of the right lower lobe. Histologic evaluation of the transbronchial biopsies revealed a mucinous adenocarcinoma (Figure 3). The patient ultimately opted for hospice care.

## Discussion

The clinical manifestations of pulmonary adenocarcinoma can be widely variable and are often dependent on the stage at which a patient presents. Low clinical-stage lung cancer typically presents with solitary lung nodules/masses. Pulmonary adenocarcinomas exist as a spectrum that can range from a precursor non-invasive lesion called atypical adenomatous hyperplasia (AAH) to a pure lepidic-type adenocarcinoma/adenocarcinoma in situ (formerly known as bronchoalveolar adenocarcinoma), to minimally invasive adenocarcinomas, to invasive adenocarcinomas. These adenocarcinomas can exist with mucinous, non-mucinous, or mixed histologies. AAH often appears radiographically as a ground glass nodule [2,3]. Pathologically, AAH would have a lepidic-type appearance but measure less than 5 mm in size. Adenocarcinoma in situ (AIS) is similarly seen as a ground glass nodule but can be part solid or contain bubble-like lucencies [2-4]. Pathologically, AIS would exist as a pure lepidic-type pattern and be greater than 5 mm in size, but less than 3.0 cm in size. Minimally invasive adenocarcinoma is distinguished by the development of part solid components in the nodule, pathologically seen as invasive non-lepidic patterns measuring less than 5 mm [2,3]. Invasive adenocarcinoma usually appears as partially solid nodules with the solid component measuring more than 5 mm [2,3]. Invasive mucinous subtypes of pulmonary adenocarcinoma are usually solid nodules but may have lobulated margins, areas of consolidation, air bronchograms, or cystic lucencies [4]. Location is also relevant. Earlier manifestations are typically peripheral in the upper lobes, while later presentations spread to multiple lobes in the bilateral lungs [2,4].

While adenocarcinoma of the lung may present with the above predictable findings, imaging can also confound the picture as there are many mimics. Benign lung cysts, blebs, or bullae appearing as cystic-like lucencies on imaging may decrease suspicion of malignancy [5]. Bronchiectasis within consolidation may also appear as cystic lucencies and can be interpreted as areas of inflammation [5]. Surveillance is important in such situations in order to monitor for progressive changes over time, as cystic lesions are rarely malignant [5]. Adenocarcinoma usually has a slower growth rate, which can sometimes imitate granulomatous disease [5]. Particularly relevant to the present case, adenocarcinoma is less likely to present as a multifocal disease with bilateral distributions [5]. More importantly, and paramount within this case, cavitary lung lesions further obscure the clinical picture as the differential diagnoses are widespread including infections, infarcts, septic emboli, rheumatic manifestations, pulmonary Langerhans cell histiocytosis, and metastases, among others. Of non-small cell lung carcinomas, cavitary lesions typically represent squamous cell carcinomas rather than adenocarcinomas [5-7].

The present case highlights a unique presentation for pulmonary adenocarcinoma, which to the best of our knowledge, has yet to have been seen in prior case reports. On review of the literature, a few case reports have been published citing adenocarcinoma of the lung presenting as cavitary lesions and with similar clinical differentials to include infection and NBTE. One such report had similar CT evidence of patchy airspace consolidations with cavitations and was also initially thought to be infectious in origin and treated with antibiotic therapy [8]. CT imaging in two other cases revealed multiple cavitary lesions similar to our present case [9,10]. Another report described CT findings of a single cavitary lesion, later pathologically confirmed as adenocarcinoma [11]. Two additional cases reported echocardiogram findings suggestive of infective endocarditis; one such case diagnosed NBTE of the mitral valve in a patient with known stage 4 malignancy [12]. Another case presented initially with acute deep venous thrombosis and pulmonary embolism with an incidentally identified lung mass; subsequently, this patient developed additional thrombotic events eventually leading to the diagnosis of NBTE of the mitral valve in the setting of large cell carcinoma [13].

It is important to bring attention to this unique clinical presentation of the present case. Primary mucinous adenocarcinoma of the lung has yet to have been recorded in the literature masquerading as suspected infective endocarditis with septic emboli to the lungs. Additionally, an echocardiogram demonstrating potential vegetations of the tricuspid valve further supported this clinical thinking, again highlighting the uniqueness of this case. The prolonged clinical course helps to illustrate the importance of knowing the many ways in which pulmonary adenocarcinoma can mimic non-malignant etiologies; presenting with bilateral lung nodules with cavitary appearances. Despite the initial concern for an infectious etiology, the patient’s clinical symptoms failed to resolve despite multiple treatments with antibiotics. This prompted further exploration of the differential diagnosis including culture-negative endocarditis, and after repeat bronchoscopies, ultimately revealing the underlying malignancy.

## Conclusions

Herein, we have described a unique case of mucinous adenocarcinoma of the lung presenting with multifocal and bilateral lung involvement. Initial clinical management was based on potential infectious etiologies, despite multiple hospitalizations with antibiotic treatment and utilization of multiple imaging modalities. As described above, lung cancer can have a diverse spectrum of radiographic presentations, both as suspicious spiculated nodules, in addition to benign appearing mimics including multifocal and bilateral cavitary lesions. Such findings emphasize the importance of short-interval clinical follow-up and serial imaging to rule out malignant etiologies.

It is paramount to consider neoplasia early in the differential diagnosis, despite these atypical clinical settings. Failure to do so can result in prolonged clinical workups with delays in the final diagnosis. We urge clinicians to consider malignancy in similar cases in the future to allow for prompt recognition and treatment.
